# Phytoplankton consortia as a blueprint for mutually beneficial eukaryote-bacteria ecosystems based on the biocoenosis of *Botryococcus* consortia

**DOI:** 10.1038/s41598-021-81082-1

**Published:** 2021-01-18

**Authors:** Olga Blifernez-Klassen, Viktor Klassen, Daniel Wibberg, Enis Cebeci, Christian Henke, Christian Rückert, Swapnil Chaudhari, Oliver Rupp, Jochen Blom, Anika Winkler, Arwa Al-Dilaimi, Alexander Goesmann, Alexander Sczyrba, Jörn Kalinowski, Andrea Bräutigam, Olaf Kruse

**Affiliations:** 1 Algae Biotechnology and Bioenergy, Faculty of Biology, Bielefeld University, Universitätsstrasse 27, 33615 Bielefeld, Germany; 2 Center for Biotechnology (CeBiTec), Bielefeld University, Universitätsstrasse 27, 33615 Bielefeld, Germany; 3 Computational Metagenomics, Faculty of Technology, Bielefeld University, Universitätsstrasse 25, 33615 Bielefeld, Germany; 4 Bioinformatics and Systems Biology, Justus-Liebig-University, Heinrich-Buff-Ring 58, 35392 Gießen, Germany; 5 Computational Biology, Faculty of Biology, Bielefeld University, Universitätsstrasse 27, 33615 Bielefeld, Germany

**Keywords:** Computational biology and bioinformatics, Genome informatics, Systems biology, Plant symbiosis, Microbial communities

## Abstract

Bacteria occupy all major ecosystems and maintain an intensive relationship to the eukaryotes, developing together into complex biomes (i.e., phycosphere and rhizosphere). Interactions between eukaryotes and bacteria range from cooperative to competitive, with the associated microorganisms affecting their host`s development, growth and health. Since the advent of non-culture dependent analytical techniques such as metagenome sequencing, consortia have been described at the phylogenetic level but rarely functionally. Multifaceted analysis of the microbial consortium of the ancient phytoplankton *Botryococcus* as an attractive model food web revealed that its all abundant bacterial members belong to a niche of biotin auxotrophs, essentially depending on the microalga. In addition, hydrocarbonoclastic bacteria without vitamin auxotrophies seem adversely to affect the algal cell morphology. Synthetic rearrangement of a minimal community consisting of an alga, a mutualistic and a parasitic bacteria underpins the model of a eukaryote that maintains its own mutualistic microbial community to control its surrounding biosphere. This model of coexistence, potentially useful for defense against invaders by a eukaryotic host could represent ecologically relevant interactions that cross species boundaries. Metabolic and system reconstruction is an opportunity to unravel the relationships within the consortia and provide a blueprint for the construction of mutually beneficial synthetic ecosystems.

## Introduction

Bacteria are omnipresent and affect likely every eukaryotic system through their presence^1^. Microalgae are the dominant primary producers and the base of the food web within aquatic ecosystems and sustain in the natural environment in close relationship with multiple associated microorganisms^2,3^. Thus, the interactions between phytoplankton and bacteria represent a fundamental ecological relationship within aquatic environments^2^, by controlling nutrient cycling and biomass production at the food web base. The nature of the exchange of micro- and macronutrients, diverse metabolites including complex products such as polysaccharides, and infochemicals defines the relationship of the symbiotic partners^4–6^, which span mutualism, commensalism and parasitism^3^. Within a parasitic association, the bacteria are known to adversely affect the algae^7^, while in commensalism the partners can survive independently, with the commensal feeding on algae-provided substrates, but without harming the host^3^. Mutualistic interactions include i.a. the obligate relationships between vitamin-synthesizing bacteria and vitamin-auxotrophic phytoplankton species^8^. Many microalgae cannot synthesize several growth-essential vitamins (like B_12_ and B_1_)^8–10^ and obtain them through a symbiotic relationship with bacteria in exchange for organic carbon^11^. Recent observations suggest that not only certain microalgae require vitamins, but many bacteria also have to compete with other organisms for the B-vitamins^12,13^.

However, in microbial environments, where the distinction between host and symbionts is less clear, the identification of the associated partners and the nature of their relationship is challenging. Looking for a suitable model food web system to elucidate the complexity of eukaryote-prokaryote interactions, we focused on the microalga-bacteria consortia with an organic carbon-rich phycosphere. The planktonic chlorophyta *Botryococcus braunii* excretes long-chain hydrocarbons and a variety of exo-polysaccharides^14^ which allows this single celled alga to form large agglomerates^1^. Both organic carbon-based products are exposed to the surrounding ecosystem and enable the alga to sustain in the natural environment (phycosphere) in close relationship with multiple associated microorganisms^2,3^. *Botryococcus* exudes up to 46% of their photosynthetically fixed carbon into the phycosphere^15^, similarly to processes, however with lesser extent, observed for seedlings as well as adult plants within the rhizosphere in the form of poorly characterized rhizodeposits^16–18^. Additionally, it is generally considered that a progenitor of *Botryococcus* impacted the development of today's oil reserves and coal shale deposits^19^. Thus, the microbiome associated with *Botryococcus braunii* presents a unique opportunity to dissect the roles of the eukaryote and prokaryotes within the consortium and test how the eukaryote responds to perturbations in its environmental context.

The interactions between *B. braunii* and the associated bacteria are not fully understood, but are known to influence the microalgal growth performance and product formation capacity^14,20–22^. The grouping of interdependent organisms living and functionally interacting with each other in the same habitat is also called biocoenosis, a term coined by Karl Möbius in 1877^23^. To understand the complex nature of the unique habitat of a microalgal phycosphere and to unravel the food web between alga and bacteria, we investigated the consortia accompanying the hydrocarbon/carbohydrate-producing green microalga *Botryococcus braunii*.

## Results

### Metagenomic survey of the *Botryococcus braunii* consortia

**Figure 1:**
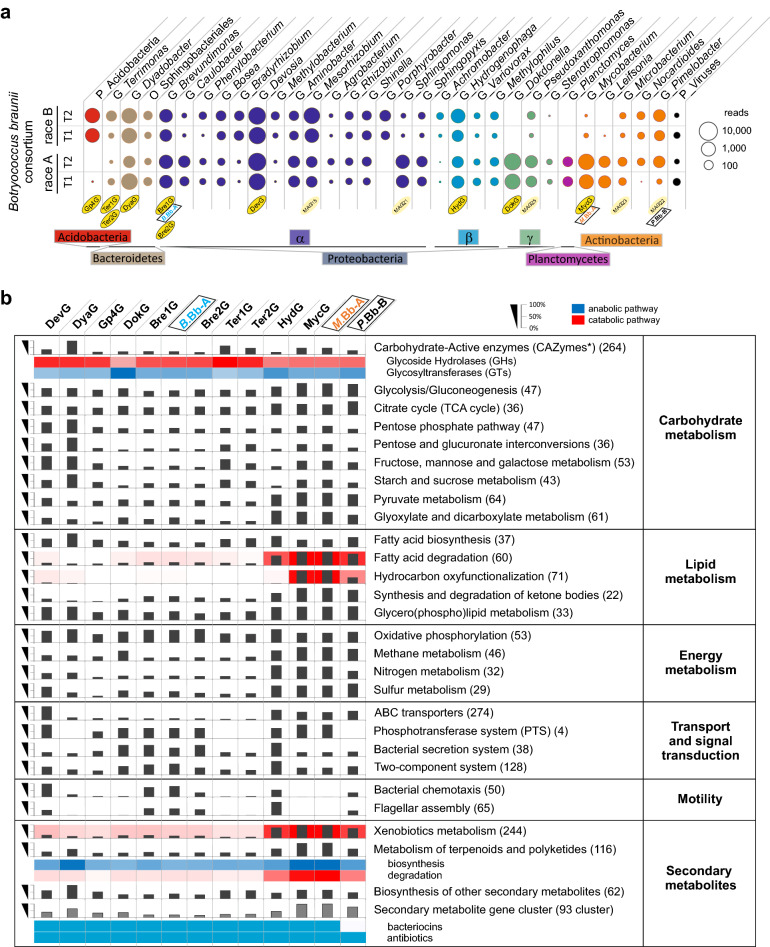
Taxonomy of the *B. braunii* bacterial community and selected functional categories encoded in reconstructed MAGs and complete genomes. (**a**) Normalized read-based comparison and taxonomic assignment of different *B. braunii* metagenome datasets. The circle size represents the amount of classified reads for the respective taxonomic group (minimum 50 reads per taxonomic group). Abbreviations: P, phylum; G, genus; O, order. (**b**) Abundance of selected functional categories encoded in the high-quality draft and complete bacterial genomes. Numbers in parentheses represent the maximal total number of genes within each pathway. Percentage: per cent of total number of genes per pathway identified within each genome. All annotated genes within the respective pathways are presented on the EMGB platform (https://emgb.cebitec.uni-bielefeld.de/Bbraunii-bacterial-consortium/). The pathways statistics are summarized in the Table S5.

To determine the inhabitants of the *B. braunii* ecosystem, we profiled four different *Botryococcus* communities (races A and B) using high-throughput 16S rDNA gene amplicon and read-based metagenome sequencing approaches (Figures S1, S2, S3; Fig. 1a). Assembly and functional annotation of the metagenomes together with single strain isolation and sequencing reconstructed the capabilities of the most abundant consortial microorganisms and were used to design and test synthetic alga-bacteria ecosystems. The analysis of the 16S rDNA amplicons and a read-based metagenome approach showed that each of the *B. braunii* communities represents a comparable conserved consortium of a single algal and at least 25 different bacterial species as well as traces of Archaea and viruses (Figure S2). In total, 16S rDNA amplicon sequencing identified 33 bacterial taxa, 22 of which were present in both race A and B consortia (Figure S3, Supplementary Discussion, Chapter 1). The relative abundance of plastid 16S rDNA sequences accounted up to 49 and 59% of all detected amplicon reads in race A and B samples, respectively. Combined metagenome de novo assembly and differential coverage and tetra-nucleotide signature based binning resulted in ten high quality and ten fragmentary bacterial metagenome assembled genomes (MAGs). Three additional genomes were closed by single strain sequencing (Fig. 1; Tables S1 and S2; Supplementary Discussion, Chapter 1). The bacterial community varied quantitatively, but not qualitatively between growth phases and strains (Figure S3). *B. braunii* consortia are inhabited by the bacterial phyla (*Proteobactreria*, *Bacteroidetes*, *Acidobacteria* and *Actinobacteria*), comparable to those occupying the plant rhizosphere^24^, albeit in different relative abundances.

During the growth phase, the *Botryococcus* phycosphere is dominated by the genera of *Devosia*, *Dyadobacter*, *Dokdonella*, *Acidobacteria*, *Brevundimonas*, *Mesorhizobium* and *Hydrogenophaga* which account for over 80% of the consortial bacteria (Table S1; Figure S4). The re-processing of the *B. braunii* Guadeloupe strain metagenome data^25^ and direct comparison of the different datasets revealed the coinciding presence of the individual bacterial genera, although massively varying in the abundance pattern in dependence of cultivation conditions (Figure S5).The same taxa are also present *i.a.* in the active microbiome associated roots and rhizosphere of oilseed rape^26^, as well as in the lettuce^27^ and global citrus rhizosphere^24^, strongly suggesting an overall mutual dependence or preferred coexistence.

To identify genes that enable the bacterial community members to contribute to and to benefit from the food web of the consortium, we performed a KEGG-based quantitative functional assignment of the annotated high-quality MAGs and the sequenced genomes of isolated associates (for details see Methods; Supplementary Discussion, Chapter 2).

We originally expected a uniform distribution of abilities and a high degree of interconnectedness between all members of the consortium. Surprisingly, the consortium divides into two functionally distinct groups of bacteria: The dominant consortia members such as *Devosia* (DevG), *Dyadobacter* (DyaG) and *Brevundimonas* (Bre1G, *B.*Bb-A) do not oxyfunctionalize hydrocarbons^28^, but their genomes are rich in genes involved in the degradation/assimilation of carbohydrates (Fig. 1b). On the contrary, the bacterial isolates *Mycobacterium* and *Pimelobacter* (*M*.Bb-A and *P*.Bb-B), which are of low abundance within the consortia (Figure S3), contain a large portfolio of genes encoding monooxygenases/hydroxylases for hydrocarbon degradation^28^ and hold the potential for a pronounced catabolic capacity towards lipids, fatty acids and xenobiotics (Fig. 1b). Besides, *Mycobacterium* and *Pimelobacter* genomes were the only genomes among the examined community members to carry non-heme membrane-associated monooxygenase *alkB* genes (Table S6)^28^. To test these observed genetic predisposition of the consortia, we assessed algal capacities for growth and hydrocarbon production in co-cultivation of the axenic *B. braunii* with one representative of each group.

### Effect of the bacterial community on the *B. braunii* host

**Figure 2:**
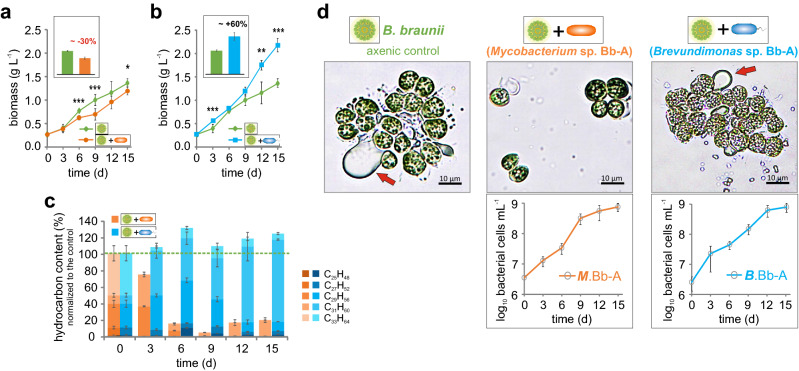
Physiological effect of the individual bacterial isolates on the microalgae during co-cultivation. (**a**–**d**) Determination of algal growth and product formation performance of axenic and xenic *B. braunii* cultures, supplemented with bacterial isolates. Shown are growth analysis of *B. braunii* in the presence of (**a**) *Mycobacterium* sp. Bb-A and (**b**) *Brevundimonas* sp. Bb-A. The inserts within the graph show the maximal observed growth difference in comparison to the control. (**c**) Comparison of the hydrocarbon levels observed in samples cultivated with bacterial isolates in relation to the axenic *B. braunii* culture (values set to 100%) in the course of the cultivation; (**d**) Morphological characteristic of algal cells during the cultivation (pictures were taken after 9 days of cultivation) with the bacterial isolates (red arrows indicate the hydrocarbons produced by the microalga). Graphs imbedded in the pictures show the progress of the bacterial growth (cell number on log_10_ scale). The error bars represent standard error of mean values of three biological and three technical replicates (SE; n = 9). Asterisks represent *p *values as determined via Student’s t-test (*=  < 0.05, **=  < 0.01, ***=  < 0.001).

One representative of the abundant as well as less abundant community members (*Brevundimonas* sp. Bb-A and *Mycobacterium* sp. Bb-A, respectively) were isolated in axenic culture and used for the co-cultivation experiments. Synthetic algae-bacteria consortia were formed with an axenic hydrocarbon- and carbohydrate producing *B. braunii* race A strain SAG30.81 under strict phototrophic conditions (*e.g.* without the supplementation of vitamins or organic carbon). Axenic *Botryococcus braunii* shows a continuous increase in cell biomass and hydrocarbon content, yielding 1.36 ± 0.1 g L^−1^ and 0.5 ± 0.1 g L^−1^, respectively (Fig. 2a, b; Figure S6). Co-cultivation of the *Brevundimonas* sp. Bb-A promotes the growth of the alga and increases the biomass by 60.5 ± 16.9% after 15 days of cultivation compared to axenic control (Fig. 2b). While co-cultivation with the *Mycobacterium* sp. Bb-A results in reduced growth of the consortia by 12.6 ± 4.1% at the end of cultivation with a maximal reduction of 30.6 ± 4.8% after 9 days (Fig. 2a).

Axenic *B. braunii* accumulates hydrocarbons up to 38% of its dry weight (DW) in a bacteria-free environment (Figure S6a), (the value was set to 100% for easier comparison in Fig. 2c). In contrast, co-cultivation with *Mycobacterium* limits hydrocarbon accumulation down to 4.4 ± 0.2% compared to the axenic control (Fig. 2c) and alters colony morphology (Fig. 2d). Loss of the hydrocarbon matrix reduces massively the colony size (Fig. 2d) and likely increases susceptibility to grazers^29^. The dramatic decrease in hydrocarbon content and disturbed colony formation cannot be observed in cultures supplemented with *Brevundimonas* or in the xenic *Botryococcus braunii* algae-bacteria communities (Fig. 2c, d; Figures S1 and S6), pointing to a balanced equilibrium between the alga and its microbiome in natural environments.

Since *Botryococcus* readily releases huge amounts of organic carbon into the extracellular milieu^15^, creating a phycosphere that naturally attracts many microorganisms, including those with potential damaging effects, its evolutionary fitness will depend on efficient management of the “bacterial zoo”. We hypothesized that there are strong control mechanisms that allow the algae host to attract and control symbiotic / beneficial bacteria. Therefore, we examined the genetic portfolio of the bacterial community for essential cofactors and interdependencies.

### The core community of *Botryococcus* phycosphere belongs to a niche of biotin auxotrophs

**Figure 3:**
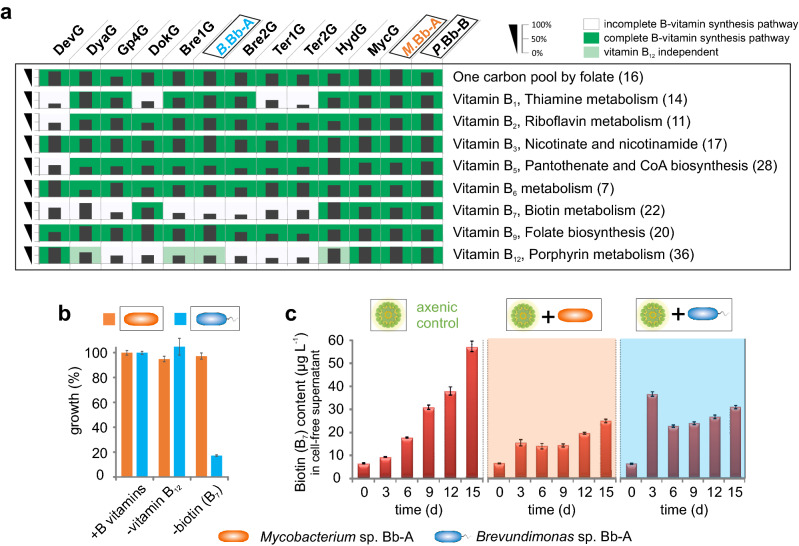
Essential cofactors (inter-)dependencies of the bacterial core community and isolates of *B. braunii*. (**a**) Heatmap of the reconstructed de novo biosynthesis of B-vitamins encoded in the high-quality draft and complete bacterial genomes. Numbers in parentheses represent the maximal total number of genes within each pathway. Percentage: per cent of total number of genes per pathway identified within each genome. All annotated genes within the respective pathways are presented on the EMGB platform (https://emgb.cebitec.uni-bielefeld.de/Bbraunii-bacterial-consortium/). The pathways statistics are summarized in the Table S5 and S8. (**b**) Growth assay to confirm B-vitamin auxotrophy and prototrophy in axenic *Brevundimonas* sp. Bb-A (blue) and *Mycobacterium* sp. Bb-A (orange) strains, respectively. (**c**) Biotin concentration levels detected in cell-free supernatants from the cultures of axenic *B. braunii* as well as in the presence of *Mycobacterium* sp. Bb-A (orange) and *Brevundimonas* sp. Bb-A (blue). The error bars represent standard error of mean values of three biological and three technical replicates (SE; n = 9).

At first glance, the *Botryococcus* alga does not appear to be obligatory dependent on a bacterial community, since axenic strains can be fully photoautotrophically cultured without vitamin supplements (Fig. 2 a, b) and contains the vitamin B_12_-independent form of methionine synthase (*metE*)^30^. (Meta)Genome reconstruction revealed that the *B. braunii* bacterial core community is auxotrophic for various B-vitamins (Fig. 3a), making them dependent on an exogenous supply of vitamins for survival. The complete thiamin synthesis pathway (vitamin B_1,_ essential for citrate cycle and pentose phosphate pathway^12^) could not be reconstructed for four assembled genomes. Five genera are auxotrophic for cobalamin (vitamin B_12_). All abundant genera within the *Botryococcus* consortia (including the growth-promoting *Brevundimonas*, (Fig. 2b)) lack the complete gene portfolio for biotin (vitamin B_7_) synthesis (Fig. 3a), but contained the complete biosynthesis pathway of fatty acids for which biotin is essential^12^. In contrast, low abundant consortial members such as *Mycobacterium* are B-vitamin prototrophs (Fig. 3a).

Extended experimental analysis confirmed the biotin (vitamin B_7_) auxotrophy in *Brevundimonas* sp. Bb-A and vitamin B prototrophy in *Mycobacterium* sp. Bb-A as deduced from the assembled genomes (Fig. 3a, b). *Botryococcus braunii* secretes and accumulates biotin into the surrounding environment (Fig. 3c). The biotin secretion appears to be induced by the presence of bacteria, in the cultures with the B_7_-auxotrophic *Brevundimonas* as well as in the presence of the biotin-prototrophic *Mycobacterium* sp. Bb-A, although to a lesser extent (cf. Figure 3c at timepoint 3 days). The subsequent decrease in biotin amount during the progress of the cultivation further hints at the active vitamin absorption from the culture supernatant in both co-cultivation approaches in comparison to the axenic control (Fig. 3c), especially during bacterial proliferation, with the biotin levels increasing again when bacterial growth stagnates (Fig. 2d, bacterial growth determination for *M*.Bb-A and *B*.Bb-A).

Collectively, in a healthy (natural) community, non-hydrocarbon degrading bacterial genera which are auxotrophic for one or more vitamins appear to dominate the *Botryococcus* phycosphere, while fully prototrophic hydrocarbonoclastic bacteria are present but not abundant. We hypothesized that *B. braunii* cultivates its own auxotrophic “bacterial zoo” to combat hydrocarbonoclastic bacteria. To test the hypothesis, synthetic consortia of alga–”good bacteria”– “bad bacteria” were formed and analyzed.

### Bacterial biotin-auxotrophic symbionts defend their eukaryotic host

**Figure 4:**
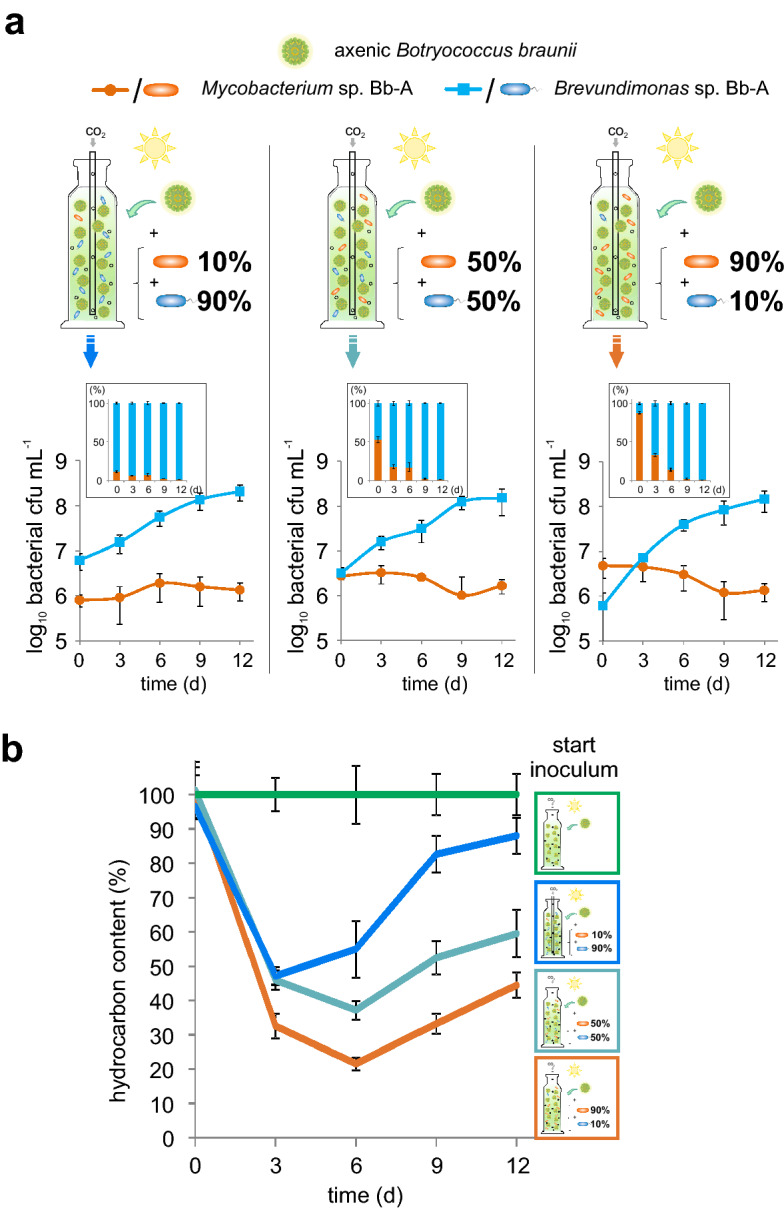
Bacterial symbionts fend their eukaryotic host from hydrocarbonoclastic invaders. Physiology of a synthetic alga-bacteria-bacteria community, consisting of *Brevundimonas* sp. Bb-A (blue) and *Mycobacterium* sp. Bb-A (orange) in co-culture with *B. braunii* (green) under photoautrophic conditions with three different inoculum ratios (10:90, 50:50 or 90:10 per cent based on *cfu* (colony-forming units)). Shown are (**a**) the progression of bacterial proliferation (*cfu*) and (**b**) detected relative hydrocarbon levels (normalized to the axenic control) during the co-cultivation. The error bars represent standard error of mean values of three biological and three technical replicates (SE; n = 9).

Based on the abovementioned findings, we have hypothesized that the alga maintains its balanced, ‘healthy' microbiome by recruiting beneficial, vitamin-auxotrophic microorganisms, *inter alia*, with the provision of photosynthetically fixed carbon and biotin, and thus counteracts pathogen assault. To test this hypothesis, one representative of each group, the vitamin-auxotrophic *Brevundimonas* and the prototroph *Mycobacterium* were co-cultivated with the axenic *Botryococcus* alga under photoautrophic conditions with three different inoculum ratios (10:90, 50:50 or 90:10 per cent based on *cfu*) (Fig. 4a).

Independently from the initial amount of inoculum, biotin-auxotrophic *Brevundimonas* sp. Bb-A proliferated strongly within this synthetic community and reached in each case a comparable *cfu*-density of up to 2.1*10^8^ cells mL^−1^. *Mycobacterium* did not proliferate in the presence of *Brevundimonas* (Fig. 4a), but was clearly capable of growth in the presence of the microalga alone (Fig. 2d). The propagation of *Mycobacterium* sp. Bb-A, limited in each approach to a maximum *cfu* of up to 1.7*10^6^ cells mL^−1^ at the end of the cultivation, is therefore likely to be attributable to the bioactive compounds with bacteriostatic properties produced by *Brevundimonas*. Indeed, all reconstructed genomes encode metabolic gene cluster for antibiotic compounds (especially against Gram-positive bacteria) and for bacteriocins (Fig. 1b, Table S9) with significant antimicrobial potency^32^. The protective effect of *Brevundimonas* appears to be further evident from the hydrocarbon content of cultures, which increases with decreasing proportions of *Mycobacterium* (Fig. 4b). Experimental analyses (Figs. 2, 3, 4) thus support key conjectures derived from metagenomic assemblies (Figs. 1 and 3a).

## Discussion

Microalgae exist in the natural environment as part of a complex microbial consortium, where the different species may exert considerable influence on each other for the exploitation of unique biological functions and/or for the metabolite exchange^2^. Bacterial communities within the *Botryococcus* phycosphere appear to be highly conserved and illustrate relatively low species biodiversity (Figures S2a and S3), with most of the detected genera occurring in all tested consortia, albeit differentially abundant (Fig. 2; Figure S5;^25^). The evaluation further emphasizes dynamic consortia^33^ that vary considerably depending on physiological conditions, especially in artificial systems (e.g. supplementation of vitamins and/or antibiotics^25^). Under strict photoautotrophic conditions, most abundant genera identified within the *Botryococcus* communities (such as *Devosia*, *Dyadobacter*, *Brevundimonas* etc.; Fig. 1a) also occur in other biomes such as phycosphere and/or rhizosphere^24,26,27,34^, strongly indicating preferential coexistence of certain bacteria and eukaryotes.

The fact that *Botryococcus* readily excretes huge amounts of their photosynthetically fixed organic carbon into its phycosphere^15^ (Figure S1) naturally attracts many microorganisms, that can be potentially harmful, so the evolutionary fitness of the algae host depends heavily on the efficient management of the "bacterial zoo", which requires strong control mechanisms that facilitate the recruitment and control of beneficial bacterial partners.

**Figure 5:**
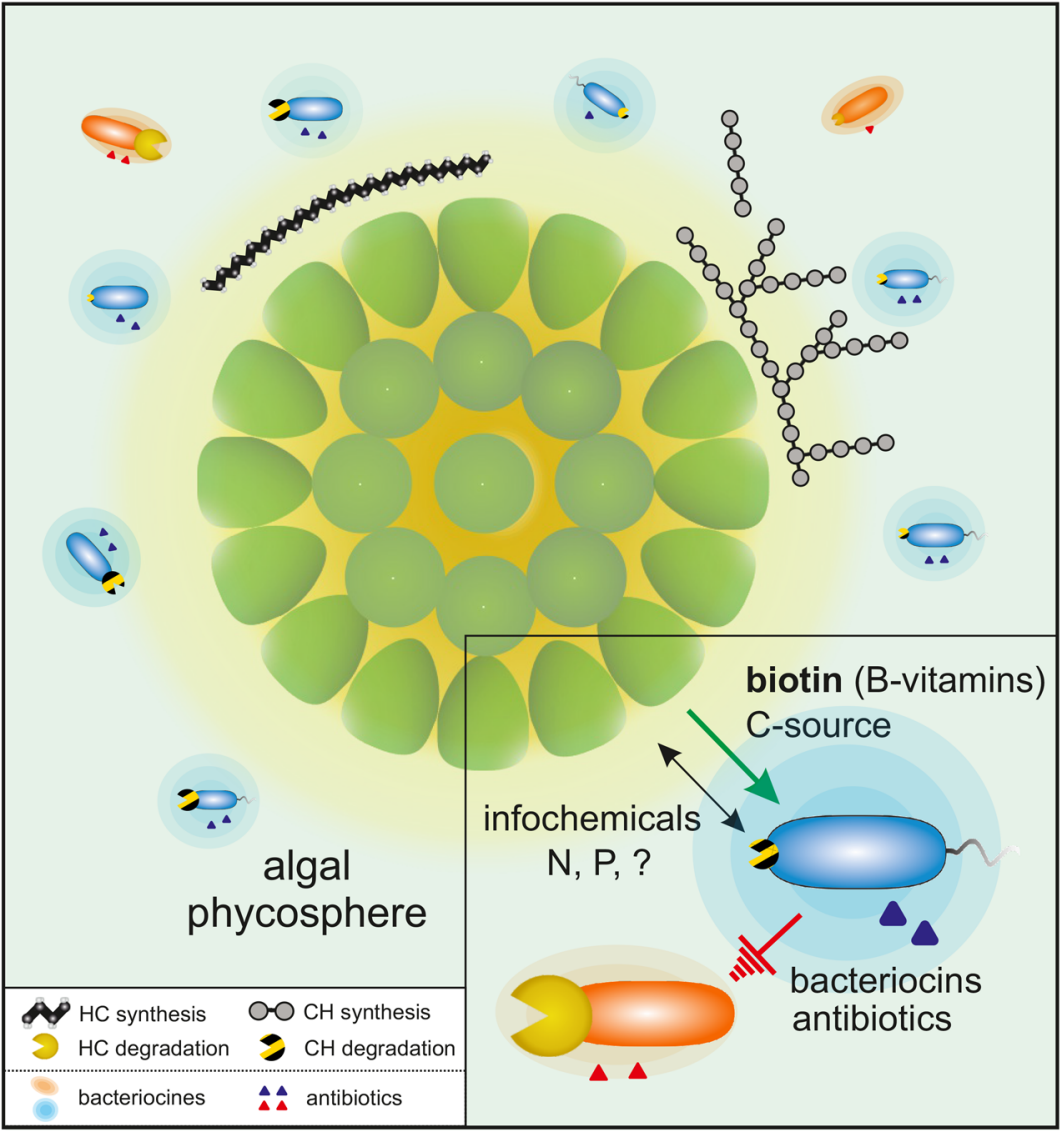
Alga-bacteria interactions within the *Botryococcus braunii* phycosphere. The depiction summarizes the observed generic potential encoded in the (meta)genomes of bacterial associates (Figs. 1 and 3), along with experimental validation within the minimal synthetic community (Figs. 2 and 4). The color code indicates the functional grouping of the community members: supportive B-vitamin-auxotrophic (blue) and hydrocarbonoclastic prototrophic (red). The potential for degradation of hydrocarbons and carbohydrates of the bacterial associates is illustrated via yellow and striped pacmans, respectively. The genetic predisposition to the synthesis of bacteriocins and antibiotics encoded by the secondary metabolite biosynthesis gene clusters, is indicated by the halos and triangles, respectively. Abbreviation: HC, Hydrocarbons; CH, carbohydrates; B_7_, Biotin; N, nitrogen; P, phosphorus.

Based on the genetically encoded features, the bacterial associates of *B. braunii* consortia can be apportioned into two functionally distinct categories of (i) B-vitamin-auxotrophic carbohydrate-degrader (controllable), and (ii) prototrophic hydrocarbonoclastic species (uncontrollable) (Fig. 5).

The first category includes the most abundant bacterial members of the *Botryococcus* consortia that do not assimilate hydrocarbons and display B-vitamin-auxotrophy, strongly depending on exogenous supply for their survival. Although, auxotrophic for various B-vitamins and non-viable on its own, the consortia’s most abundant species *Devosia* (DevG, up to 30% abundancy of all bacteria, Table S3) is the only representative of the first category to contain the complete pathway for vitamin B_12_ synthesis (Fig. 3a). In view of the fact that the bacterial abundance determines their functional relevance for the community^35,36^, this consortia-dominant species (DevG) is likely to provide both microalga and bacteria with the essential co-factor^8^, thus supporting the recent proposition that the B_12_ supply is realized only by a few certain bacteria within the alga-bacteria consortia^34^. The release of vitamins and vitamin precursors by bacteria is a well known mediator of algae-bacteria interactions^9,33^, where the transfer of vitamins like cobalamin and thiamin^8,37^ supports algal growth and can be considered as ‘bacterial farming’ of algae as providers of organic resources. However, our observations show that *B. braunii* does not essentially depend on microbial vitamin supply. Further, according to the (meta)genome reconstruction results, the *Botryococcus* bacterial consortia cultivated under strictly phototrophic conditions is dominated by vitamin-auxotrophs (Fig. 3a). Especially the biotin-auxotrophic bacteria are prevalent within the *Botryococcus* consortia, so it is doubtful that the large required supply of biotin is of bacterial origin. Because of its predominance within the community (Figure S2) and the observed active biotin secretion (Fig. 3c), the microalgae tend to be the main source for the consortial biotin supply. The excretion of some B-vitamins has been observed in microalgae and plants^38,39^, however the increasingly induced vitamin secretion by the presence of bacteria, as well as their simultaneous active uptake, remained so far unrecognized. This observation supports the recent proposition that many microorganisms although capable of vitamin synthesis themselves if needed, likely prefer to take up the compounds when available in the environment, because synthesis requires usually more metabolic energy than transport^31^. However, we cannot rule out that the lower level of induction of biotin secretion observed for the biotin-prototrophic *Mycobacterium* sp. Bb-A (Fig. 3C), may result from a possible suppression of biotin formation in *B. braunii* by the bacterium or the recognition of the bacterium as a non-symbiont by the algae. Nevertheless, since some of the missing cofactors may only be complemented by bacteria (e.g. vitamin B_12_), strong bacteria-bacteria relations within the consortia occur in addition to the algae-bacteria interactions, signifying a deep degree of interconnectedness within the *Botryococcus* phycosphere.

Completely autonomous species, such as the isolate *Mycobacterium* sp. Bb-A are rare among the consortia and assigned to the second category (uncontrollable). These phylotypes are known to be hydrocarbonoclastic microorganisms^40,41^ targeting mainly the organic carbon source and therefore pose a potential risk to the microalga, strongly implying a parasitic relationship. And although these phylotypes are able to provide the *Botryococcus* alga with vitamin B_12_, nutrients and probiotic compounds, their activity results in a less complex colony structure due to the degradation of the hydrocarbon matrix (Fig. 2). The observed change of the colony morphology might affect the survival of the typically colonized microalgae in the natural environment, i.e. by higher susceptibility to grazers^29^. The malfunction of colony formation could thus be attributed to imbalanced and dysbiotic host-microbiome interaction similar to imbalanced interactions previously observed^42,43^, so that prototrophic, mainly carbon-targeting bacterial species may essentially act as pathogens.

In this context, it seems reasonable to assume that specific bacteria that require essential co-factors are deliberately promoted and controlled by *Botryococcus braunii* in exchange for protection as well as nutrients and probiotic metabolites. The observed growth-promoting effect of *Brevundimonas* (Fig. 2b), which depends on exogenous biotin supply for survival (Fig. 3), is thus based on the close interaction with the microalga. This mutualistic alga-bacteria relationship seems efficiently to prevent uncontrolled overgrowing of hydrocarbonoclastic competitors, as demonstrated by the experiments with the minimal synthetic consortia (Fig. 4).

Thus, the interactions between the microalga and bacteria within the *Botryococcus braunii* consortia depends on a complex interplay between all members, with the microalga as the “conductor” in this play (Fig. 5). However, the elucidation of the microalga-bacteria dynamics as well as of the communication signals exchanged between the participating parties requires more precise kinetic analyses and will be subject for further research. In this context, the metagenomic analyses of the *Botryococcus* consortium might represent a path towards mechanistic resolution of ecological interactions at the microscopic scale.

## Materials and methods

### Strains, cultivation conditions and growth determination parameters

Liquid, xenic cultures of *Botryococcus braunii* race A (CCALA778, isolated in 1997 in Serra da Estrela, Baragem da Erva da Fome, Portugal) and race B (AC761, isolated in 1983 from Paquemar, Martinique, France) as well as the axenic *B. braunii* race A strain (SAG30.81, Peru, Dpto.Cuzco, Laguna Huaypo) were used for co-cultivation studies. The cultures were phototrophically cultivated at room temperature (~ 22 °C) under 16:8 light–dark illumination of 350 ± 400 μmol photons m^−2^ s^−1^ white light in modified Chu-13 media (without the addition of vitamin and organic carbon source) as described before^15^. Carbon supply and agitation were achieved by bubbling the cultures with moisture pre-saturated carbon dioxide-enriched air (5%, v/v) with a gas flow rate of 0.05 vvm. Cultures growth was monitored by measurements of biomass organic dry weight (DW) by drying 10 mL culture per sample (in three biological and technical replicates) following the protocol described before^44^. Additionally, microalgae and bacteria cells were regularly checked by optical microscopy.

The bacterial isolates were obtained by plating highly diluted xenic *B. braunii* cultures (CCALA778 and AC761) on LB agar plates supplemented with vitamins (0.1 nM cobalamin, 3.26 nM thiamine and 0.1 nM biotin, Sigma Aldrich). After 1–2 weeks single cell colonies were picked, separately plated and analyzed via 16S rDNA Sanger-sequencing by using the universal primer pair 27F (5′-AGAGTTTGATCCTGGCTCAG-3′) and 1385R (5′-CGGTGTGTRCAAGGCCC-3′). Two isolates, deriving from algal race A and one from race B, were classified based on 16S rDNA similarity as *Mycobacterium*, *Brevundimonas* and *Pimelobacter* species, respectively. The purity of the axenic alga and bacterial strains was regularly verified by polymerase chain reaction (PCR) amplification and Sanger-sequencing of the 16S rRNA gene using a universal primer pair 27F and 1385R. All primers were purchased by Sigma Aldrich.

For experiments testing the requirement of B-vitamins, mono-cultures of *Mycobacterium* sp. Bb-A and *Brevundimonas* sp. Bb-A were pre-washed three times in minimal medium^45^ and then starved of vitamins for two days prior to the start of the experiment. Then, bacterial cells were diluted to 0.2 (oD_600_) with minimal medium supplemented with 1gL^−1^ glucose and B-vitamins (at the following concentrations: 0.1 nM (each) of cobalamin, riboflavin, biotin and pantothenate as well as 3.26 nM thiamine, Sigma Aldrich) and grown for seven days.

For the co-cultivation experiments, 500 mL axenic algal cultures (~ 0.25 g L^−1^) were inoculated with the isolates (*Mycobacterium* sp. Bb-A or *Brevundimonas* sp. Bb-A, with ~ 2 × 10^6^ cells mL^−1^ for single isolate cultivation and with ~ 2 × 10^6^–2 × 10^7^ cells mL^−1^ (in different ratios 10:90, 50:50 and 90:10 (*cfu*/*cfu*)) for co-cultivation with both bacterial strains) and cultured under photoautotrophic conditions. The bacterial cell density was determined by manual cell counting using hemocytometer or by determination of the colony-forming units (*cfu*) as a proxy for viable population density using the replica plating method^46^.

The quantitative determination of the biotin concentration in cell-free supernatants of the co-cultures and the axenic control was accomplished by using microbiological microtiter plate test (VitaFast, r-biopharm, Darmstadt, Germany) according to manufacturer instructions.

### Hydrocarbon extraction and analysis

Hydrocarbon extraction was performed according to the protocol published previously^15^. The dry extracted hydrocarbons were resuspended in 500 µL of *n*-hexane, containing an internal standard *n*-hexatriacontane (C_36_H_74_, Sigma Aldrich) and analysed via GC–MS and GC-FID as described before^15,47^.

### DNA sample collection and preparation

The samples from xenic *B. braunii* race A and B cultures (three biological replicates) were taken in the linear and stationary growth phases (after 9 and 24 days). Total community genomic DNA was extracted as previously described^48^. The quality of the DNA was assessed by gel electrophoresis and the quantity was estimated using the Quant-iT PicoGreen dsDNA Assay Kit (Invitrogen) and a Tecan Infinite 200 Microplate Reader (Tecan Deutschland GmbH).

### High-throughput 16S rDNA amplicon sequencing

To get insight into the community composition of *B. braunii* Race A and B, high-throughput 16S rDNA amplicon sequencing was performed as described recently by Maus et al^49^. The primer pair Pro341F (5′-CCTACGGGGNBGCASCAG-3′) and Pro805R (5′-GACTACNVGGGTATCTAATCC-3′) was used to amplify the hypervariable regions V3 and V4 of diverse bacterial and archaeal 16S rRNAs^50^. In addition, the primers also cover the 16S rDNA gene of algal chloroplasts and eukaryotic mitochondrial genomes. Furthermore, in a two PCR steps based approach, multiplex identifier (MID) tags and Illumina-specific sequencing adaptors were added to each amplicon. Only amplicons featuring a size of ~ 460 bp were purified using AMPureXP magnetic beads (Beckman Coulter GmbH, Brea, California, USA). Resulting amplicons were qualitatively and quantitatively analyzed using the Agilent 2100 Bioanalyzer system (Agilent Inc., Santa Clara, California, USA) and pooled in equimolar amounts for paired-end sequencing on the Illumina MiSeq system (Illumina, San Diego, California USA). This sequencing approach provided ~ 150,000 reads per sample. An in-house pipeline as described previously^51^ was used for adapter and primer trimming of all samples. For amplicon processing, a further in house amplicon processing pipeline including FLASH^52^, USEARCH^53^ (v8.1), UPARSE^54^ and the RDP^55^ classifier (v2.9) was applied as described recently^56,57^. In summary, unmerged sequences resulting by FLASH ^52^ (default settings + −M 300) were directly filtered out. Furthermore, sequences with > 1Ns (ambiguous bases) and expected errors > 0.5 were also discarded. Resulting data was further processed and operational taxonomic units (OTUs) were clustered by applying USEARCH^53^ (v8.1). The resulting OTUs were taxonomically classified using the RDP^55^ classifier (v2.9) in 16S modus (Threshold > 0.8) and compared to the nt database by means of BLASTN^58^. In the last step, raw sequence reads were mapped back onto the OTU sequences in order to get quantitative assignments.

### (Meta)Genome sequencing, assembly, binning and annotation

For each of the four samples (*B. braunii* race A and B from linear (T1) and stationary (T2) growth phase), the genomic DNA from three replicates was pooled. To obtain the metagenome sequence, four whole-genome-shotgun PCR-free sequencing libraries (Nextera DNA Sample Prep Kit; Illumina, Munich, Germany) were generated based on the manufacturer’s protocol representing different time points of the cultivation and different algae communities. The libraries were sequenced in paired-end mode in a MiSeq run (2 × 300 bp) resulting in 11,963,558 and 11,567,552 reads for race A T1 and T2; 6,939,996 and 12,031,204 reads for race B T1 and T2, respectively (total 12.75 Gb). The de novo metagenome assembly was performed using the Ray Meta assembler^59^ (v2.3.2) using a k-mer size of 41 and default settings. For contigs with length larger than 1 kb (n = 127,914), the total assembly size was 338.5 Mb, the N50 = 2884 and the largest contig 1.59 Mb. All processed raw sequence reads were aligned to the assembled metagenome contigs by means of Bowtie 2^60^ (v2.2.4). By using SAMtools^61^ (v1.6), the SAM files were converted to BAM files. These files were sorted and read mapping statistics were calculated. The following portions of reads could be assembled to the draft metagenomes: 70.1% and 77.5% for race A (T1 and T2); 51.0% and 62.9% for race B (T1 and T2). For binning of the metagenome assembled genomes (MAGs), the tool MetaBAT (v0.21.3)^62^ was used with default settings. Completeness and contamination of the resulting MAGs were tested with BUSCO^63,64^ (v3.0.), using the Bacteria and Metazoa data set (Table S1).

For sequencing of the isolated species, 4 µg of purified chromosomal DNA^48^ was used to construct PCR-free sequencing library and sequenced applying the paired protocol on an Illumina MiSeq system, yielding ~ 1.71 Mio Reads (517 Mb in 3 Scaffolds, 58 Contigs) for *Mycobacterium* sp. Bb-A and ~ 1.70 Mio Reads (503 Mb in 6 Scaffolds, 65 Contigs) for *Pimelobacter* sp. Bb-B. Obtained sequences were de novo assembled using the GS de novo Assembler software (v2.8, Roche). An in silico gap closure approach was performed as described previously^65^. MinION sequencing library with genomic DNA from *Brevundimonas* sp. Bb-A was prepared using the Nanopore Rapid DNA Sequencing kit (SQK-RAD04) according to the manufacturer's instructions with the following changes: The entry DNA amount was increased to 800 ng and an AMPure XP bead cleanup was carried out after transposon fragmentation. Sequencing was performed on an Oxford Nanopore MinION Mk1b sequencer using a R9.5 flow cell, which was prepared according to the manufacturer's instructions. MinKNOW (v1.13.1) was used to control the run using the 48 h sequencing run protocol; base calling was performed offline using albacore (v2.3.1). The assembly was performed using canu v1.7^66^, resulting in a single, circular contig (170 Mb, 1 kb to 17.5 kb). This contig was then polished with Illumina short read data from the metagenome data set using pilon^67^, run for sixteen iterative cycles. bwa-mem^68^ was used for read mapping in the first eight iterations and bowtie2 v2.3.2^60^ in the second set of eight iterations. Annotation of the draft chromosomes was performed within the PROKKA^69^ (v1.11) pipeline and visualized using the GenDB 2.0 system^70^.

### Gene prediction, taxonomic assignment and functional characterization

Gene prediction on the assembled contigs of the MAGs was performed with Prodigal^71^ (v2.6.1) using the metagenome mode (“-p meta”). All genes were annotated and analyzed with the in-house EMGB annotation system using the databases NCBI NR^58^, Pfam^72^ and KEGG^73^. Based on DIAMOND^74^ (v0.8.36) hits against the NCBI-NR^58^ database, all genes were subject to taxonomic assignment using MEGAN^75^. Relationships to metabolic pathways were assigned based on DIAMOND^74^ hits against KEGG^73^. For Pfam^72^ annotations, pfamscan was used with default parameters. The genomes were manually inspected using an E-value cutoff of 1 × 10^–10^ for several specific sequences listed in Additional file 2, Table S6. The profiling of the carbohydrate-active enzymes (CAZy) encoded by the *B. braunii* community members was accomplished using dbCAN2^76^ metaserver (Table S7). The identification, annotation and analysis of the secondary metabolite biosynthesis gene clusters within the MAGs and complete genomes were accomplished via the antiSMASH^77^ (v4.0) web server with default settings (Table S9).

### Phylogenetic analyses

To assign and phylogenetically classify the MAGs, a core genome tree was created by applying EDGAR^78^ (v2.0). For calculation of a core-genome-based phylogenetic tree, the core genes of all MAGs and the three isolates *Mycobacterium* sp. Bb-A, *Brevundimonas* sp. Bb-A and *Pimelobacter* sp. Bb-B, corresponding to selected reference sequences were considered. The implemented version of FASTtree^79^ (using default settings) in EDGAR^78^ (v2.0) was used to create a phylogenetic Maximum-Likelihood tree based on 53 core genes (see Table S4) of all genomes. Additionally, the MAGs were taxonomically classified on species level by calculation of average nucleic and amino acid identities (ANI, AAI) by applying EDGAR^78^ (v2.0). For phylogenetic analysis of the 16S rDNA amplicon sequences, MEGA^80^ (v7.0.26) was used. Phylogenetic tree was constructed by means of Maximum-Likelihood Tree algorithm using Tamura 3-parameter model. The robustness of the inferred trees was evaluated by bootstrap (1000 replications).

## Supplementary Information

Below is the link to the electronic supplementary material.

Supplementary Information 1.

Supplementary Information 2.

## Data Availability

The high-throughput 16S rDNA amplicon sequencing datasets obtained during the present work are available at the EMBL-EBI database under BioProjectID PRJEB21978. The chloroplastic 16S rRNA genes sequencing data of *B. braunii* SAG 30.81 are available under the GenBank accession number MN956340. The metagenome datasets of *Botryococcus braunii* consortia as well as the resulting MAGs have been deposited under the BioProjectIDs PRJEB26344 and PRJEB26345, respectively. The genomes of the isolates *Brevundimonas* sp. Bb-A, *Mycobacterium* sp. BbA or *Pimelobacter* sp. Bb-B are accessible under BioProjectIDs PRJNA528993, PRJEB28031 und PRJEB28032, respectively. In addition, the communities most abundant ten high-quality draft as well as isolate genomes are also accessible via EMGB platform (https://emgb.cebitec.uni-bielefeld.de/Bbraunii-bacterial-consortium/).
